# Supplementary material for: Identification of photoperiod-induced specific miRNAs in the adrenal glands of Sunite sheep (Ovis aries)
Source: Front Vet Sci. 2022 Jul 22;9:888207. doi: 10.3389/fvets.2022.888207 (PMC9354845; doi:10.3389/fvets.2022.888207)
Supplement: Supplementary file 1 [file Data_Sheet_1.ZIP › Supplementary file/miRDeep2 file.zip!/miRDeep2 file/Know_Structure/LP42A2/oar-let-7b.pdf]

miRBase precursor : oar-let-7b
Total read count : 184417
oar-let-7b read count : 183543
remaining reads : 874

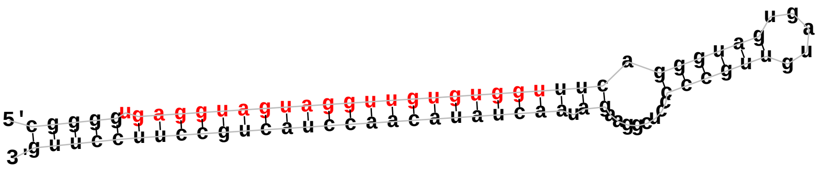

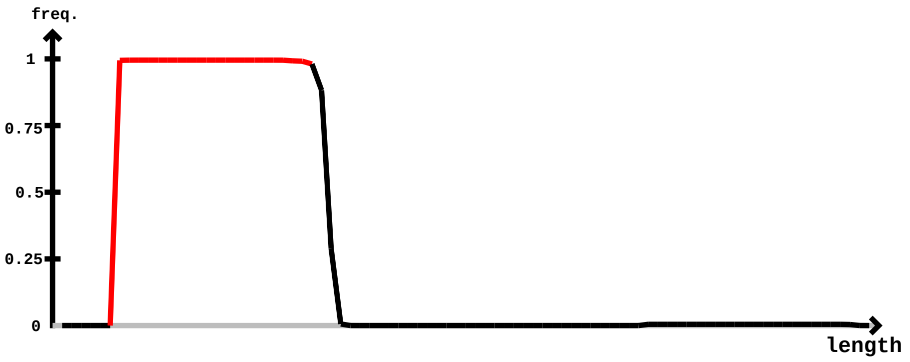


```
      oar-let-7b
5'- cggggugagguaguagguuguguggu uucaggguagugauguugcccccucggaagauaacuauacaaccuacugccuuccuug   -3'    exp
    (((((.(((((((((((((((((((((((.((((((.....))))))(....)..)).))))))))))))))))))))))))))    reads      mm         sample
    ....gugagguaguagguugugugguuu........................................................    1          0          seq
    .....ugagguaguagguugug..............................................................    30         0          seq
    .....ugagguaguagguugugu.............................................................    453        0          seq
    .....ugagguaguagguugugug............................................................    282        0          seq
    .....ugagguaguagguugugugg...........................................................    1896       0          seq
    .....ugagguaguagguuguguggu..........................................................    18163      0          seq
    .....ugagguaguagguugugugguu.........................................................    109402     0          seq
    .....ugagguaguagguugugugguuu........................................................    52076      0          seq
    .....ugagguaguagguugugugguuuc.......................................................    1092       0          seq
    .....ugagguaguagguugugugguuuca......................................................    13         0          seq
    ......gagguaguagguugugugg...........................................................    3          0          seq
    ......gagguaguagguuguguggu..........................................................    11         0          seq
    ......gagguaguagguugugugguu.........................................................    89         0          seq
    ......gagguaguagguugugugguuu........................................................    25         0          seq
    ......gagguaguagguugugugguuuc.......................................................    2          0          seq
    .......agguaguagguugugugguu.........................................................    2          0          seq
    .......agguaguagguugugugguuu........................................................    2          0          seq
    ........gguaguagguugugugguu.........................................................    1          0          seq
    .............................aggguagugauguugcccccucggaagauaa........................    1          0          seq
    ......................................auguugcccccucggaagaua.........................    1          0          seq
    .........................................uugcccccucggaagauaacu......................    1          0          seq
    ..............................................cccucggaagauaacuauacaaccu.............    1          0          seq
    .........................................................uaacuauacaaccuacugccuuc....    1          0          seq
    .........................................................uaacuauacaaccuacugccuucc...    1          0          seq
    ..........................................................aacuauacaaccuacugccuuc....    1          0          seq
    ..........................................................aacuauacaaccuacugccuucc...    1          0          seq
    ...........................................................acuauacaaccuacugccuuccu..    1          0          seq
    ............................................................cuauacaaccuacugccu......    1          0          seq
    ............................................................cuauacaaccuacugccuuc....    9          0          seq
    ............................................................cuauacaaccuacugccuucc...    112        0          seq
    ............................................................cuauacaaccuacugccuuccu..    710        0          seq
    ............................................................cuauacaaccuacugccuuccuu.    28         0          seq
    .............................................................uauacaaccuacugccuuccu..    4          0          seq
    ..............................................................auacaaccuacugccuuccu..    1          0          seq
```
